# First cases of coronavirus disease 2019 (COVID-19) in the WHO European Region, 24 January to 21 February 2020

**DOI:** 10.2807/1560-7917.ES.2020.25.9.2000178

**Published:** 2020-03-05

**Authors:** Gianfranco Spiteri, James Fielding, Michaela Diercke, Christine Campese, Vincent Enouf, Alexandre Gaymard, Antonino Bella, Paola Sognamiglio, Maria José Sierra Moros, Antonio Nicolau Riutort, Yulia V. Demina, Romain Mahieu, Markku Broas, Malin Bengnér, Silke Buda, Julia Schilling, Laurent Filleul, Agnès Lepoutre, Christine Saura, Alexandra Mailles, Daniel Levy-Bruhl, Bruno Coignard, Sibylle Bernard-Stoecklin, Sylvie Behillil, Sylvie van der Werf, Martine Valette, Bruno Lina, Flavia Riccardo, Emanuele Nicastri, Inmaculada Casas, Amparo Larrauri, Magdalena Salom Castell, Francisco Pozo, Rinat A. Maksyutov, Charlotte Martin, Marc Van Ranst, Nathalie Bossuyt, Lotta Siira, Jussi Sane, Karin Tegmark-Wisell, Maria Palmérus, Eeva K. Broberg, Julien Beauté, Pernille Jorgensen, Nick Bundle, Dmitriy Pereyaslov, Cornelia Adlhoch, Jukka Pukkila, Richard Pebody, Sonja Olsen, Bruno Christian Ciancio

**Affiliations:** 1 European Centre for Disease Prevention and Control, Stockholm, Sweden; 2 World Health Organisation Regional Office for Europe, Copenhagen, Denmark; 3 Robert Koch Institute, Berlin, Germany; 4 Santé Publique France – Direction des maladies infectieuses, Saint-Maurice, France; 5 Centre national de référence Virus des infections respiratoires, dont la grippe, Institut Pasteur, Paris, France; 6 Centre national de référence Virus des infections respiratoires, dont la grippe, Hospices civils de Lyon, Lyon, France; 7 Istituto Superiore di Sanita, Rome, Italy; 8 Istituto Nazionale Malattie Infettive Lazzaro Spallanzani, Rome, Italy; 9 Coordination Centre for Health Alerts and Emergencies. Spanish Ministry of Health, Madrid, Spain; 10 Servicio de Epidemiología, Dirección General de Salut Pública, Islas Baleares, Spain; 11 Federal Service for Surveillance on Consumer Rights Protection and Human Well-being (Rospotrebnadzor), Moscow, Russia; 12 Department of Infectious Disease Prevention and Control, Common Community Commission, Brussels-Capital Region, Brussels, Belgium; 13 Chief Physician, Infection control unit, Lapland Hospital District, Rovaniemi, Finland; 14 County Medical Officer, Jönköping Region, Jönköping, Sweden; 15 Santé publique France – Direction des régions, Cellule régionale Nouvelle Aquitaine, Bordeaux, France; 16 Santé publique France – Direction des régions, Cellule régionale Ile-de-France, Paris, France; 17 Santé publique France – Direction des régions, Cellule régionale Auvergne-Rhône-Alpes, Lyon, France; 18 National Centre for Microbiology, WHO-National Influenza Centre, Institute of Health Carlos III. Madrid, Spain; 19 National Centre of Epidemiology, CIBERESP, Institute of Health Carlos III. Madrid, Spain; 20 Dirección General de Salut Pública, Islas Baleares, Spain; 21 State Research Center of Virology and Biotechnology “Vector”, Rospotrebnadzor, Moscow, Russia; 22 St. Pierre Hospital, Brussels, Belgium; 23 Laboratory of Clinical Virology, Department of Microbiology and Immunology, Rega Institute, KU Leuven - University of Leuven, Leuven, Belgium; 24 Epidemiology of infectious diseases, Sciensano, Brussels, Belgium; 25 Expert Microbiology Unit, Department of Health Security, Finnish Institute for Health and Welfare (THL), Helsinki, Finland; 26 Infectious Disease Control and Vaccinations Unit, Department of Health Security, Finnish Institute for Health and Welfare (THL), Helsinki, Finland; 27 Public Health Agency of Sweden, Solna, Sweden; 28 Jönköping Region, Jönköping, Sweden; 29 These authors have contributed equally to the manuscript

**Keywords:** COVID-19, Novel coronavirus, SARS-COV-2, SARS, coronavirus disease 2019

## Abstract

In the WHO European Region, COVID-19 surveillance was implemented 27 January 2020. We detail the first European cases. As at 21 February, nine European countries reported 47 cases. Among 38 cases studied, 21 were linked to two clusters in Germany and France, 14 were infected in China. Median case age was 42 years; 25 were male. Late detection of the clusters’ index cases delayed isolation of further local cases. As at 5 March, there were 4,250 cases.

A cluster of pneumonia of unknown origin was identified in Wuhan, China, in December 2019 [1]. On 12 January 2020, Chinese authorities shared the sequence of a novel coronavirus termed severe acute respiratory syndrome coronavirus 2 (SARS-CoV-2) isolated from some clustered cases [2]. Since then, the disease caused by SARS-CoV-2 has been named coronavirus disease 2019 (COVID-19). As at 21 February 2020, the virus had spread rapidly mostly within China but also to 28 other countries, including in the World Health Organization (WHO) European Region [3-5]. Here we describe the epidemiology of the first cases of COVID-19 in this region, excluding cases reported in the United Kingdom (UK), as at 21 February 2020. The study includes a comparison between cases detected among travellers from China and cases whose infection was acquired due to subsequent local transmission.

## Surveillance in the WHO European Region

On 27 January 2020, the European Centre for Disease Prevention and Control (ECDC) and the WHO Regional Office for Europe asked countries to complete a WHO standard COVID-19 case report form for all confirmed and probable cases according to WHO criteria [6-8]. The overall aim of surveillance at this time was to support the global strategy of containment of COVID-19 with rapid identification and follow-up of cases linked to affected countries in order to minimise onward transmission. The surveillance objectives were to: describe the key epidemiological and clinical characteristics of COVID-19 cases detected in Europe; inform country preparedness; and improve further case detection and management. Data collected included demographics, history of recent travel to affected areas, close contact with a probable or confirmed COVID-19 case, underlying conditions, signs and symptoms of disease at onset, type of specimens from which the virus was detected, and clinical outcome. The WHO case definition was adopted for surveillance: a confirmed case was a person with laboratory confirmation of SARS-CoV-2 infection (ECDC recommended two separate SARS-CoV-2 RT-PCR tests), irrespective of clinical signs and symptoms, whereas a probable case was a suspect case for whom testing for SARS-CoV-2 was inconclusive or positive using a pan-coronavirus assay [8]. By 31 January 2020, 47 laboratories in 31 countries, including 38 laboratories in 24 European Union and European Economic Area (EU/EEA) countries, had diagnostic capability for SARS-CoV-2 available (close to 60% of countries in the WHO European Region), with cross-border shipment arrangements in place for many of those lacking domestic testing capacity. The remaining six EU/EEA countries were expected to have diagnostic testing available by mid-February [9].

## Epidemiology of first cases in the European Region

As at 09:00 on 21 February 2020, 47 confirmed cases of COVID-19 were reported in the WHO European Region and one of these cases had died [4]. Data on 38 of these cases (i.e. all except the nine reported in the UK) are included in this analysis.

The first three cases detected were reported in France on 24 January 2020 and had onset of symptoms on 17, 19 and 23 January respectively [10]. The first death was reported on 15 February in France. As at 21 February, nine countries had reported cases (Figure): Belgium (1), Finland (1), France (12), Germany (16), Italy (3), Russia (2), Spain (2), Sweden (1) and the UK (9 – not included further).

**Figure:**
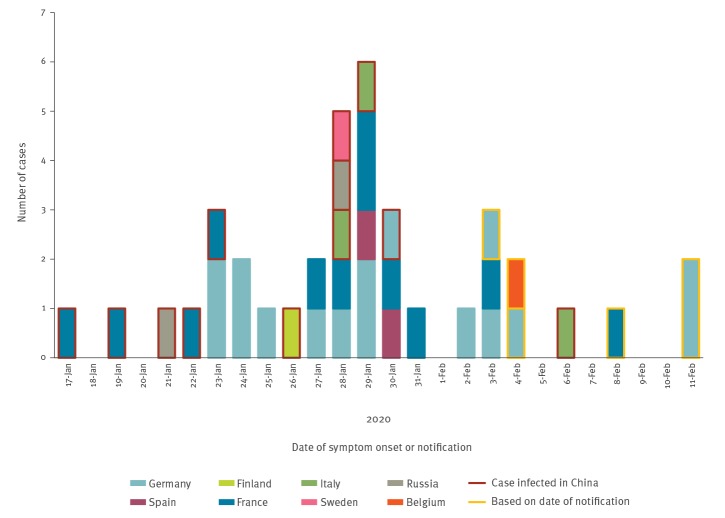
Epidemic curve of reported COVID-19 cases by date of symptom onset, or date of notification, WHO European Region, as at 21 February 2020 (n = 38)^a,b^

COVID-19: coronavirus disease 2019; WHO: World Health Organization.

^a^ Four cases were reported by Germany with unknown date of onset and unknown symptomatology. Belgium and France reported one asymptomatic case each. These cases are displayed according to their date of notification.

^b^ The figure does not include nine cases reported in the United Kingdom.

The place of infection (assessed at national level based on an incubation period presumed to be up to 14 days [11], travel history and contact with probable or confirmed cases as per the case definition) was reported for 35 cases (missing for three cases), of whom 14 were infected in China (Hubei province: 10 cases; Shandong province: one case; province not reported for three cases). The remaining 21 cases were infected in Europe. Of these, 14 were linked to a cluster in Bavaria, Germany, and seven to a cluster in Haute-Savoie, France [12,13]. Cases from the Bavarian cluster were reported from Germany and Spain, whereas cases from the Haute-Savoie cluster were reported from France and Spain. Cases linked to the Haute Savoie cluster were also detected in the UK, including the index case of this cluster, who was infected in Singapore before travelling to France [14]. The index cases for the cluster in Bavaria was reported to be infected in China [15].

The median age of the 38 cases was 42 years (range: 2–81 years) and 25 were male (Table). The proportion of male cases was higher among cases acquired in Europe (14 males of 21 cases) compared with those acquired in China (8 males of 14 cases) although the difference was not statistically significant (chi-squared test: p = 0.6). There was no difference in median age by sex (males: 45 years; females: 38 years, k-sample median test, p = 1.0) or by whether infection was acquired in Europe or not (acquired in Europe: 47 years; acquired in China: 38 years, p = 0.2).

**Table:** Characteristics of confirmed COVID-19 cases, WHO European Region, as at 21 February 2020 (n = 38)^a^

Characteristics	Number of cases overall	Number of cases infected in Europe	Number of cases infected in China	p value^b^
Age range in years				
0–17	4	3	0	0.08
18–49	24	13	9	
50–64	7	5	2	
≥ 65	3	0	3	
Sex				
Male	25	14	8	0.6
Female	13	7	6	
Hospitalised				
Yes	35	21	14	ND
No	2	ND	ND	
Unknown	1	ND	ND	
Symptoms^c ^(31 cases with available information)				
Asymptomatic	2	ND	ND	ND
Fever	20	ND	ND	
Cough	14	ND	ND	
Weakness	8	ND	ND	
Headaches	6	ND	ND	
Sore throat	2	ND	ND	
Rhinorrhoea	2	ND	ND	
Shortness of breath	2	ND	ND	
Mean days from onset to hospitalisation (29 cases)	3.7	4.6	2.5	ND
Mean days from onset to first positive laboratory test (16 cases)	5.1	6.5	5.2	ND

COVID-19: coronavirus disease 2019; ND: not determined or known; WHO: World Health Organization.

^a^ The Table does not include nine cases reported in the United Kingdom.

^b^ Chi-squared test for difference in distributions between cases infected in Europe and those infected in China.

^c^ A single case could have more than one symptom. Only symptoms reported by two or more cases shown.

All but two cases were hospitalised (35 of 37 where information on hospitalisation was reported), although it is likely that most were hospitalised to isolate the person rather than because of severe disease. The time from onset of symptoms to hospitalisation (and isolation) ranged between 0 and 10 days with a mean of 3.7 days (reported for 29 cases). The mean number of days to hospitalisation was 2.5 days for cases imported from China, but 4.6 days for those infected in Europe. This was mostly a result of delays in identifying the index cases of the two clusters in France and Germany. In the German cluster, for example, the first three cases detected locally were hospitalised in a mean of 5.7 days, whereas the following six took only a mean of 2 days to be hospitalised.

Symptoms at the point of diagnosis were reported for 31 cases. Two cases were asymptomatic and remained so until tested negative. The asymptomatic cases were tested as part of screening following repatriation and during contact tracing respectively. Of the remaining 29, 20 reported fever, 14 reported cough and eight reported weakness. Additional symptoms reported included headaches (6 cases), sore throat (2), rhinorrhoea (2), shortness of breath (2), myalgia (1), diarrhoea (1) and nausea (1). Fever was reported as the sole symptom for nine cases. In 16 of 29 symptomatic cases, the symptoms at diagnosis were consistent with the case definition for acute respiratory infection [16], although it is possible that cases presented additional symptoms after diagnosis and these were not reported. 

Data on pre-existing conditions were reported for seven cases; five had no pre-existing conditions while one was reported to be obese and one had pre-existing cardiac disease. No data on clinical signs e.g. dyspnea etc. were reported for any of the 38 cases.

All hospitalised cases had a benign clinical evolution except four, two reported in Italy and two reported in France, all of whom developed viral pneumonia. All three cases who were aged 65 years or over were admitted to intensive care and required respiratory support and one French case died. The case who died was hospitalised for 21 days and required intensive care and mechanical ventilation for 19 days. The duration of hospitalisation was reported for 16 cases with a median of 13 days (range: 8–23 days). As at 21 February 2020, four cases were still hospitalised.

## Laboratory diagnosis

All cases were confirmed according to specific assays targeting at least two separate genes (envelope (E) gene as a screening test and RNA-dependent RNA polymerase (RdRp) gene or nucleoprotein (N) gene for confirmation) [8,17]. The specimen types tested were reported for 27 cases: 15 had positive nasopharyngeal swabs, nine had positive throat swabs, three cases had positive sputum, two had a positive nasal swab, one case had a positive nasopharyngeal aspirate and one a positive endotracheal aspirate.

## Discussion

As at 09:00 on 21 February, few COVID-19 cases had been detected in Europe compared with Asia. However the situation is rapidly developing, with a large outbreak recently identified in northern Italy, with transmission in several municipalities and at least two deaths [18]. As at 5 March 2020, there are 4,250 cases including 113 deaths reported among 38 countries in the WHO European region [19].

In our analysis of early cases, we observed transmission in two broad contexts: sporadic cases among travellers from China (14 cases) and cases who acquired infection due to subsequent local transmission in Europe (21 cases). Our analysis shows that the time from symptom onset to hospitalisation/case isolation was about 3 days longer for locally acquired cases than for imported cases. People returning from affected areas are likely to have a low threshold to seek care and be tested when symptomatic, however delays in identifying the index cases of the two clusters in France and Germany meant that locally acquired cases took longer to be detected and isolated. Once the exposure is determined and contacts identified and quarantined (171 contacts in France and 200 in Germany for the clusters in Haute-Savoie and Bavaria, respectively), further cases are likely to be rapidly detected and isolated when they develop symptoms [15,20]. In the German cluster, for example, the first three cases detected locally were hospitalised in a mean of 5.7 days, whereas the following six were hospitalised after a mean of 2 days. Locally acquired cases require significant resources for contact tracing and quarantine, and countries should be prepared to allocate considerable public health resources during the containment phase, should local clusters emerge in their population. In addition, prompt sharing of information on cases and contacts through international notification systems such as the International Health Regulations (IHR) mechanism and the European Commission’s European Early Warning and Response System is essential to contain international spread of infection.

All of the imported cases had a history of travel to China. This was consistent with the epidemiological situation in Asia, and supported the recommendation for testing of suspected cases with travel history to China and potentially other areas of presumed ongoing community transmission. The situation has evolved rapidly since then, however, and the number of countries reporting COVID-19 transmission increased rapidly, notably with a large outbreak in northern Italy with 3,089 cases reported as at 5 March [18,19]. Testing of suspected cases based on geographical risk of importation needs to be complemented with additional approaches to ensure early detection of local circulation of COVID-19, including through testing of severe acute respiratory infections in hospitals irrespectively of travel history as recommended in the WHO case definition updated on 27 February 2020 [21].

The clinical presentation observed in the cases in Europe is that of an acute respiratory infection. However, of the 31 cases with information on symptoms, 20 cases presented with fever and nine cases presented only with fever and no other symptoms. These findings, which are consistent with other published case series, have prompted ECDC to include fever among several clinical signs or symptoms indicative for the suspected case definition.

Three cases were aged 65 years or over. All required admission to intensive care and were tourists (imported cases). These findings could reflect the average older age of the tourist population compared with the local contacts exposed to infection in Europe and do not allow us to draw any conclusion on the proportion of severe cases that we could expect in the general population of Europe. Despite this, the finding of older individuals being at higher risk of a severe clinical course is consistent with the evidence from Chinese case series published so far although the majority of infections in China have been mild [22,23].

This preliminary analysis is based on the first reported cases of COVID-19 cases in the WHO European Region. Given the small sample size, and limited completeness for some variables, all the results presented should be interpreted with caution.

With increasing numbers of cases in Europe, data from surveillance and investigations in the region can build on the evidence from countries in Asia experiencing more widespread transmission particularly on disease spectrum and the proportion of infections with severe outcome [22]. Understanding the infection-severity is critical to help plan for the impact on the healthcare system and the wider population. Serological studies are vital to understand the proportion of cases who are asymptomatic. Hospital-based surveillance could help estimate the incidence of severe cases and identify risk factors for severity and death. Established hospital surveillance systems that are in place for influenza and other diseases in Europe may be expanded for this purpose. In addition, a number of countries in Europe are adapting and, in some cases, already using existing sentinel primary care based surveillance systems for influenza to detect community transmission of SARS-CoV-2. This approach will be used globally to help identify evidence of widespread community transmission and, should the virus spread and containment no longer be deemed feasible, to monitor intensity of disease transmission, trends and its geographical spread.

Additional research is needed to complement surveillance data to build knowledge on the infectious period, modes of transmission, basic and effective reproduction numbers, and effectiveness of prevention and case management options also in settings outside of China. Such special studies are being conducted globally, including a cohort study on citizens repatriated from China to Europe, with the aim to extrapolate disease incidence and risk factors for infection in areas with community transmission. Countries together with ECDC and WHO, should use all opportunities to address these questions in a coordinated fashion at the European and global level.
